# Surveillance of suicidal behaviour by the Belgian Network of Sentinel General Practices: trends over two decades

**DOI:** 10.1186/2049-3258-73-S1-P40

**Published:** 2015-09-17

**Authors:** Nicole Boffin, Sarah Moreels, Viviane Van Casteren

**Affiliations:** 1 Scientific Institute of Public Health, Brussels, Belgium

## Objectives

Drawing on new (2011-12) and previous (1993-5, 2000-1 and 2007-8) data on suicidal behaviour reported by the Belgian Network of Sentinel General Practices (SGP), we describe trends in the key characteristics of suicidal events. We also examine patient age-related trends in the on-site attendance of sentinel general practitioners (GPs) as first professional caregivers following suicidal behaviour and the accuracy of suicide incidence estimates. Outcome measures: Patient age and gender, suicide methods, whether the GP was the first caregiver on-site, whether the patient was new, and the outcome of the suicidal behaviour (fatal or not) were recorded on standard registration forms. The accuracy of suicide incidence estimates was tested against suicide mortality statistics.

## Results

Over the four time periods, 1671 suicidal events were reported: 275 suicides, 1287 suicide attempts and 109 events of suicidal behaviour of unknown outcome. In 2011-12, sentinel GPs' on-site attendance following the suicidal behaviour of patients < 65 years had continued to decrease (from 71% in 1993-5 to 58% in 2000-1, 39% in 2007-8 and 25% in 2011-12). In 2011-12, it had also decreased steeply in the population = 65 years (from 70% in 1993-5, 76% in 2000-1 and 79% in 2007-8 to 35% in 2011-12). No significant differences were found between the SGP-based suicide incidence estimates for 2011-2 and the available suicide mortality rates for people < 65 years and = 65 years

## Conclusions

GPs' on-site attendance as first professional caregivers following suicidal behaviour continues to decline, since 2011-2 also in the population = 65 years. Unawareness of patients' suicidal behaviour endangers both care for surviving patients and the completeness of SGP surveillance data. Yet, the incidence of suicide for 2011-12 was estimated accurately by the SGP.

